# Supplementary material for: MetaRibo-Seq measures translation in microbiomes
Source: Nat Commun. 2020 Jun 29;11:3268. doi: 10.1038/s41467-020-17081-z (PMC7324362; doi:10.1038/s41467-020-17081-z)
Supplement: Supplementary file 10 — Supplementary Data 7 [file 41467_2020_17081_MOESM10_ESM.zip › File2/Confidence_VeryHigh_Taxonomy/82911_out.krona.html]

Javascript must be enabled to view this page.

members
magnitude
magnitudeUnassigned
count
unassigned
taxon
rank

82911\_out

18
1

SRS021496\_contig\_number\_10079

4
superkingdom
2759

4751
4
kingdom

4
subkingdom
451864

4890
phylum
4

subphylum
4
147538

147550
4
class

222544
4
subclass

4
order
639021

81093
4
family

4
genus
148303

4
species
148305

SRS011115\_contig\_number\_contig-100\_2309.274597.274597SRS049253\_contig\_number\_contig-100\_1528.34437SRS078733\_contig\_number\_contig-100\_30283.106787SRS893379\_contig\_number\_contig-100\_2205.130371

12
superkingdom
2

phylum
12
1239

class
7
91061

6
order
1385

genus
6
1378

species
6

SRS011152\_contig\_number\_34080SRS011247\_contig\_number\_5170SRS011247\_contig\_number\_contig-100\_109.58293SRS013506\_contig\_number\_3144SRS013825\_contig\_number\_1154SRS014126\_contig\_number\_contig-100\_20.20
1379

1
order
186826

1300
family
1

1301
1
genus

species
1

SRS014473\_contig\_number\_7065
2069309

186801
4
class

4
order
186802

31979
family
2

1485
2
genus


SRS014888\_contig\_number\_31093
2293009
species
1

1
species
1492

SRS014573\_contig\_number\_40389

2
family
186803

genus
1
572511


SRS016969\_contig\_number\_9447
40520
species
1

genus
1
1164882


SRS056896\_contig\_number\_23209
979627
species
1

1737404
1
class

1737405
1
order

1
genus
165812

species
1

SRS011243\_contig\_number\_6700
165813


SRS057355\_contig\_number\_contig-100\_4483.88200
1
